# Neural correlates of emotion acceptance and suppression in borderline personality disorder

**DOI:** 10.3389/fpsyt.2022.1066218

**Published:** 2023-01-10

**Authors:** Silvia Carvalho Fernando, Thomas Beblo, Agnes Lamers, Nicole Schlosser, Friedrich G. Woermann, Martin Driessen, Max Toepper

**Affiliations:** ^1^Department of Psychiatry and Psychotherapy, Evangelisches Klinikum Bethel, University of Bielefeld, Bielefeld, Germany; ^2^Epilepsy Center Bethel, Mara Hospital, University of Bielefeld, Bielefeld, Germany

**Keywords:** fMRI, borderline personality disorder (BPD), emotion acceptance, emotion suppression, insula, caudate

## Abstract

**Background:**

Emotion dysregulation is a central feature of borderline personality disorder (BPD). Since impaired emotion regulation contributes to disturbed emotion functioning in BPD, it is crucial to study underlying neural activity. The current study aimed at investigating the neural correlates of two emotion regulation strategies, namely emotion acceptance and suppression, which are both important treatment targets in BPD.

**Methods:**

Twenty-one women with BPD and 23 female healthy control participants performed an emotion regulation task during functional magnetic resonance imaging (fMRI). While watching fearful movie clips, participants were instructed to either accept or to suppress upcoming emotions compared to passive viewing.

**Results:**

Results revealed acceptance-related insular underactivation and suppression-related caudate overactivation in subjects with BPD during the emotion regulation task.

**Conclusion:**

This is a first study on the neural correlates of emotion acceptance and suppression in BPD. Altered insula functioning during emotion acceptance may reflect impairments in emotional awareness in BPD. Increased caudate activity is linked to habitual motor and cognitive processes and therefore may accord to the well-established routine in BPD patients to suppress emotional experiences.

## 1. Introduction

Emotion dysregulation is a core feature of borderline personality disorder (BPD). According to Linehan’s biosocial theory (1), individuals with BPD have a heightened emotional sensitivity, are unable to regulate intense emotional responses, and return to emotional baseline more slowly than healthy persons. Based on the conceptualization of BPD as emotion dysregulation disorder, Linehan developed the Dialectal Behavior Therapy (DBT) that aims at improving emotion regulation skills and distress tolerance. Consistent with her view of BPD, there is growing evidence that impaired emotion regulation decisively contributes to emotion dysfunction in BPD. Data from several studies suggest that individuals with BPD overuse emotion regulation strategies, such as emotion suppression, that are considered to be less effective at reducing negative affect (2). In addition, BPD patients seem to underuse emotion regulation strategies, e.g., emotion acceptance, that are stated to be more effective at modifying affective outcomes. Emotion acceptance is a core element of DBT (3) and describes engaging in present-moment awareness accompanied with an openness toward feelings and emotional states without trying to control, change, suppress or avoid them (4). Emotion acceptance is often contrasted to emotion suppression and some authors consider both, at least on a conceptual level, as opposing strategies (5). Even if emotion suppression initially referred only to the expressive suppression of emotional reactions (6), emotion suppression has become a collective term referring to the rejection or reduction of any emotional experiences due to an unwillingness to experience negative emotions along with related thoughts and sensations (2). In general, higher levels of emotion acceptance and lower levels of emotion suppression are linked to reduced psychopathology and negative affectivity (7, 8). In agreement with these findings, a recent meta-analysis on the habitual use of emotion regulation strategies in BPD confirmed that higher BPD symptom severity was associated with a more intense use of emotion suppression and a less frequent use of emotion acceptance (2).

Despite these consistent findings, there has been little agreement on the effects of instructed emotion acceptance and suppression in BPD patients. In healthy controls (HC), emotion acceptance was consistently demonstrated to have beneficial effects, including reduced reports of negative affect and decreased physiological arousal in response to aversive emotions (9–12). By contrast, emotion suppression was shown to have paradoxical effects on physiological arousal in HC along with a subsequent heightened negative affect (11). For individuals with BPD, however, some previous studies suggested a link between emotion acceptance and higher subjective distress as well as increased urge for self-injury [e.g., (13, 14)]. In addition, emotion suppression was shown to provide some (at least short term) positive effects in BPD patients (e.g., reduced urges for self-injury behavior) (15).

Neuroimaging research may help to elucidate underlying neural processing associated with emotion acceptance and suppression in BPD compared to non-BPD individuals. Functional imaging studies have tried to identify the neural correlates of emotion processing in BPD and provide growing evidence that BPD patients show an increased activation of limbic brain regions including the amygdala (16) and the insular cortex (17) during the processing of negative stimuli compared to neutral conditions. This limbic hyperactivation in BPD differed from the activation patterns reported for healthy individuals and has been repeatedly associated with abnormal prefrontal brain activation in response to emotionally challenging material (18). Focusing on the impact of emotion acceptance, a recent meta-analysis of experimental fMRI studies in non-clinical samples revealed increased brain activation for acceptance compared to control (no-regulation) conditions, which involved the inferior frontal gyrus, the anterior insula, the putamen, the frontal pole and the medial prefrontal cortex/anterior cingulate cortex (4). With respect to emotion suppression, most neuroimaging studies have focused on expressive suppression, so that there is much less information about the effects of suppression of the subjective emotional experiences itself. However, a recent meta-analysis by Schulze et al. (19) provides some evidence for enhanced activation in the ventrolateral prefrontal cortex (VLPFC) during negative affective processing, which in the authors’ view could reflect a regulatory mechanism to deal with excessive affective responding. Indeed, the VLPFC was previously associated with the suppression of emotional responses (20).

To our knowledge, no previous studies have investigated the effect of instructed emotion acceptance on affective processing in BPD using fMRI. Focusing on the habitual use of emotion acceptance and suppression, a previous study of our own working group revealed hyperactivation in frontostriatal brain regions (i.e., left superior frontal gyrus, right caudate) as well as in the left precuneus, left precentral gyrus, left posterior cingulate cortex and left hippocampus when confronted with fearful (vs. neutral) film stimuli (21). The most important finding was the inverse association between striatal activation during the processing of fearful facial stimuli and the habitual disposition to accept unpleasant emotional states in BPD patients. The current study extends these findings by investigating the effects of instructed use of emotion regulation strategies in patients with BPD using a comparable experimental design. Hence, the aim of our study was to determine the neural correlates of instructed emotion acceptance and suppression compared to a non-regulation condition (passive viewing) while watching fearful cinematic sequences. As emotion acceptance and suppression are likely to be distinct emotion regulation strategies, the focus of the current study lies on strategy-specific activity patterns and not on overlapping neural activity. We expected that emotion acceptance and suppression would have divergent effects on neural response to fearful film stimuli in BPD. In line with Lamers et al. (21), we hypothesized an increased activation of the fronto-limbic-striatal emotion-related network (i.e., amygdala, insula, hippocampus, ACC, caudate, putamen, DLPFC, und VLPFC) in BPD compared to the HC group when they were instructed to suppress emotions. In contrast, we hypnotized that emotion acceptance would reduce fronto-limbic-striatal neural activation. Given the very heterogeneous previous findings on instructed emotion acceptance and suppression, it is difficult to generate specific hypotheses regarding activation differences between BPD and HC, but we exploratory investigated whether there were significant differences in the neural response to fearful stimuli between BPD and HC when applying both strategies.

## 2. Materials and methods

### 2.1. Participants

In the current work, we included 21 right-handed women with BPD and 23 female HC matched for age, handedness and intelligence. BPD patients were recruited at the University Hospital for Psychiatry and Psychotherapy, Evangelisches Klinikum Bethel, Germany. HC were recruited *via* local advertisements. All participants underwent diagnostic assessments including the Structured Clinical Interview for DSM-IV Axis-I disorders (SCID-I) (22) and the BPD section of the SCID for Axis-II disorders (SCID-II) (23). All patients met the DSM-IV criteria of BPD as assessed by experienced and trained clinical psychologists. Since our study focused on affective processing, all BPD patients additionally had to meet the diagnostic criterion of affective instability (DSM-IV-criterion 6). HC were required to have no current or past Axis I or Axis II disorders and no history of psychiatric or psychotherapeutic treatment. General exclusion criteria for all participants were current or previous severe medical conditions or CNS relevant somatic diseases, neurological disorders, a history of head injury, and conditions incompatible with MRI-investigations (e.g., metal-implants). Additional exclusion criteria for BPD patients were comorbid alcohol or drug dependence within the last 6 months, current or lifetime psychosis, current or lifetime bipolar disorder, current major depression, current eating disorder with a body mass index <15, intake of benzodiazepine, changes of psychotropic medication within the last 14 days, and DBT treatment within the last 6 months prior to study participation.

The sample consisted of Caucasian, German-speaking participants. On average, patients fulfilled 6.7 DSM-IV criteria for BPD (SD = 1.16) and 81.0% had one or more psychiatric hospitalizations in the past (M = 2.71, SD = 3.78). Fifty-seven percent of the BPD patients (*n* = 12) met criteria for one or more current comorbid Axis I disorder. The most frequent comorbid diagnosis was PTSD (*n* = 7), followed by anxiety disorders (*n* = 6), bulimia nervosa (*n* = 1), OCD (*n* = 1) and comorbid dysthymia (*n* = 1). Fourteen BPD patients (66.7%) took psychotropic mediation(s): selective serotonin reuptake inhibitors (*n* = 5), selective serotonin and norepinephrine reuptake inhibitors (*n* = 3), selective noradrenalin and dopamine reuptake inhibitors (*n* = 1), other antidepressants (*n* = 1), neuroleptics (*n* = 3), and anticonvulsants (*n* = 2). All participants provided written informed consent and received monetary compensation for taking part in the study. The study was conducted in agreement with the Declaration of Helsinki and approved by the University of Münster, Ethics Committee.

### 2.2. Psychometric measures and neurocognitive tests

The Borderline Symptom List-23 (BSL-23) (24) was applied to assess self-reported borderline symptoms. Habitual emotion acceptance and emotion suppression were measured with the Emotion Acceptance Questionnaire (EAQ) (25). Emotion regulation difficulties (i.e., non-acceptance of negative emotions, difficulties engaging in goal-directed behavior, impulse control difficulties, limited access to effective emotion regulation strategies, lack of emotional awareness, lack of emotional clarity) were assessed by mean values of the Difficulties in Emotion Regulation Scale (DERS) (26). Cognitive flexibility and executive functions were tested using a German verbal fluency task (Regensburg Word Fluency Test/RWT) (27) and the Trail-Making-Test part B (TMT-B) (28).

### 2.3. Emotion regulation task

In the current study, we used a modified and previously validated version of the fearful face paradigm (29, 30) to investigate neural activation during instructed emotion regulation, namely emotion acceptance and suppression. During fMRI scanning, participants watched 18 movie sequences with actors expressing intense fear in a pseudo-randomized order. While watching these movie sequences, participants were instructed to either suppress upcoming emotions (emotion suppression), to accept upcoming emotions (emotion acceptance) or to passively watch the movie sequences (within-subjects design). Standardized instructions to suppress or to accept upcoming emotions were adapted from Campbell-Sills and colleagues (9). To ensure that all study participants were familiar with the instructed emotion regulation strategies, they completed a 30 min training session on the emotion regulation task prior to fMRI scanning. The training session included nine practical trials with three trials per experimental condition. Fearful movie clips shown in the training session were not part of the later scanning session but comparable in valence and arousal to those used in the fMRI task.

### 2.4. Experimental procedure

**FIGURE 1:**
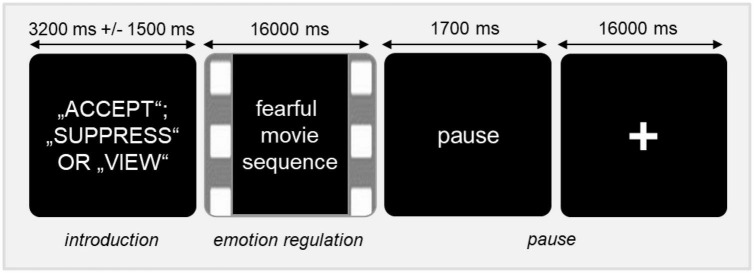
Schematic illustration of the experimental design.

After completing the training session, BPD patients and HC participants performed the emotion regulation task during fMRI. The fMRI protocol consisted of 42 experimental trials and was adapted from our previous research (21). Figure 1 illustrates the experimental design of the present study. Each trial began with the presentation of one of the three instructions (i.e., “SUPPRESS,” “ACCEPT,” OR “VIEW”) for 3,200 ms with a variable jitter between 1,700 and 4,700 ms (31). Subsequently, the participants watched movie clips for 16,000 ms, while they were instructed to regulate their feelings according to the given strategy. Thereafter, the prompt to end the regulation phase was displayed for 1,700 ms followed by a recovery phase lasting 16,000 ms.

### 2.5. Data acquisition

Functional MRI data were collected at Mara Hospital, Bethel Epilepsy Center, using a 3T Siemens Verio scanner with a quantum gradient and a standard 12-channel head coil (Siemens, Erlangen, Germany). Functional images were acquired using a single T2-weighted gradient echo planar imaging sequence with the following parameters: slice thickness 4 mm (1 mm gap), repetition time (TR) 2,100 ms, echo time (TE) 30 ms, flip angle 90^°^, field of view (FOV) 192 × 192 mm, matrix size 64 × 64, voxel size 3 × 3 × 4 mm. The number of volumes was 377, each containing 30 axial slices covering the whole brain and measured in descending order parallel to the hippocampus. Using the software Presentation^^®^^ (Neurobehavioral Systems Inc., Berkeley, CA, USA), visual stimuli were displayed on a screen inside the MRI scanner, which the participants saw through a mirror attached to the head coil. Before the EPI sequence, field map sequences were applied to control for magnetic field inhomogeneities. In addition, high-resolution anatomical images were acquired using a T1-weighted, 3-dimensional magnetization-prepared rapid gradient echo sequence (slice thickness 0.8 mm, TR 1,900 ms, TE 2.5 ms, inversion time 900 ms, flip angle 9^°^, FOV 240 × 240 mm, matrix size 320 × 320, voxel size 075 × 0.75 × 0.75 mm, 192 slices).

### 2.6. Data analysis

Demographic and questionnaire data were analyzed with independent two-sample *t*-tests using IBM SPSS Statistics version 25.0 (IBM Crop, Armonk, NY, USA). All levels of significance were α < 0.05. Imaging data were preprocessed and analyzed using SPM12 (Wellcome Centre for Human Neuroimaging, London, UK)^1^. The first three images of every EPI recording session were excluded from data analysis to ensure a stable magnetic field. The preprocessing comprised movement and slice time correction, co-registration of the T1-weighted image to the mean EPI, spatial normalization into the Montreal Neurological Institute (MNI) reference space with a 12-parameter non-linear transformation (3 × 3 × 3 mm^3^), and spatial smoothing using an isotropic 3-dimensional Gaussian kernel with a full width at half-maximum (FWHM) of 9 mm. Functional imaging data were analyzed using a general linear model with seven regressors, including one combined regressor for the instructions, one for each experimental condition (“SUPPRESS,” “ACCEPT,” OR “VIEW”), and three regressors for the pause periods, respectively. In addition, we included six head movement regressors into the design matrix as covariates of no interest to control for movement artifacts. First-level analyses involved brain activation (or deactivation) during emotional acceptance and suppression of fearful movie sequences contrasted to brain activation associated with passive viewing (contrasts reflecting activation: acceptance > passive viewing, suppression > passive viewing; contrasts reflecting deactivation: passive viewing > acceptance, passive viewing > suppression). At the second level, we conducted whole-brain analyses using within- and between-groups *t*-tests on these contrasts to identify instruction- and group-related differences in brain activation. Whole-brain results were tested at the cluster level using a threshold of *Z* > 3.1 with a minimum cluster size of *k* ≥ 20 voxels and a cluster significance threshold of *p* < 0.05, family- wise error (FWE)-corrected for multiple comparisons. In addition, we conducted regions of interest (ROI) analyses in *a priori* defined regions being related to emotion regulation. These regions included the amygdala, insula, hippocampus, ACC, caudate, putamen as well as DLPFC (BA9/46) und VLPFC (BA 44/45) (16, 19, 20). To account for possible laterality effects on emotion regulation processes (32), ROIs were analyzed for each hemisphere separately. The corresponding ROI masks were taken from the automated anatomic labelling atlas (AAL), which is implemented in the Wake Forest University (WFU) PickAtlas, an automated software toolbox for generating ROI masks based on the Talairach Daemon database. For ROI-analyses, only findings significant at *p* < 0.05 (FWE-corrected) at the voxel level were reported. To further specify significant group differences, we additionally extracted the mean signal intensity values for each significant ROI, hemisphere and participant separately by using the SPM Marsbar toolbox. Signal intensity values were then fed into 2 (emotion regulation condition) × 2 (hemispheres) × 2 (group) repeated measures ANOVAs comparing the groups with respect to signal intensity in the experimental and baseline conditions in both hemispheres.

## 3. Results

### 3.1. Demographic, psychometric, and neurocognitive data

As expected, subjects with BPD revealed significantly higher scores on the BSL-23 compared to the HC group (Table 1). Moreover, BPD patients and HC showed significant discrepancies in self-reports with regard to habitual emotion regulation. Table 2 presents descriptive statistics and group comparisons related to habitual emotion regulation as measured by the EAQ and DERS. In the EAQ, participants with BPD reported decreased emotion acceptance and increased emotion suppression compared to HC. Similarly, subjects with BPD reported significantly more emotion regulation difficulties, as measured by the DERS, than healthy participants. Given that psychotropic medication may affect emotion regulation, we performed a subgroup analysis of BPD patients to compare patients treated with psychotropic medication to unmedicated BPS individuals. We found no significant difference in self-reported habitual emotion regulation between both groups (*p* > 0.49). Furthermore, participants with BPD and HC did not differ significantly with respect to age and cognitive performance as measured by TMT and RWT.

**TABLE 1:** Demographic, neurocognitive, and psychometric data of individuals with borderline personality disorder (BPD) and healthy control participants.

	BPD Mean (SD)	HC Mean (SD)	*t (df)*	*p (two-tailed)*	*d*
Age	25.71 (6.87)	24.83 (7.83)	–0.40 (42)	0.693	–
BSL-23	1.93 (0.55)	0.31 (0.25)	12.34 (27.41)	0.014	–3.85
RWT phonemic fluency score	23.14 (6.03)	22.83 (6.54)	–0.17 (42)	0.869	–
RWT semantic fluency score	21.10 (4.16)	22.70 (4.16)	1.28 (42)	0.209	–
TMT-B (s)^a^	70.96 (31.47)	59.83 (29.99)	–1.19 (41)	0.244	–
EAQ–acceptance	2.83 (0.75)	4.19 (0.88)	5.52 (42)	<0.01	–1.66
EAQ–suppression^b^	2.48 (0.77)	4.18 (0.96)	6.51 (41.33)	<0.01	–1.94
DERS (total score)	126.80 (14.89)	67.42 (11.77)	14.54 (41)	<0.01	–4.45

BPD, borderline personality disorder; HC, healthy controls; SD, standard deviation; *d*, Cohens’ *d*; RWT, regensburg word fluency test; TMT, trail making test; EAQ, emotion acceptance questionnaire; DERS, difficulties in emotion regulation scale. ^a^Data missing for one patient. ^b^The scoring is reversed such that higher EAQ values indicate low emotion suppression.

**TABLE 2:** Results of the whole-brain analyses indicating acceptance-related deactivation of the right insula and suppression-related deactivation of the right cerebellum and the left calcarine in subjects with borderline personality disorder (BPD) during the emotion regulation task.

Brain regions	MNI coordinates			Cluster size		
	*x*	*y*	*z*	*k*	*t*	*p*
**Acceptance (acceptance < passive viewing)**						
**BPD**						
Right insula	48	–7	4	135	4.75	0.036
**HC**						
No significantly activated voxels.						
**Suppression (suppression < passive viewing)**						
**BPD**						
Right cerebellum Left calcarine	15 –9	–55 –85	–14 10	551 –	5.20 –	<0.001 –
**HC**						
No significantly activated voxels.						

BPD, borderline personality disorder; HC, healthy controls; *t*, *t*-value, *k*, cluster size (in voxels). Whole-brain results were tested at the cluster level using a threshold of *Z* > 3.1 with a minimum cluster size of *k* ≥ 20 voxels and a cluster significance threshold of *p* < 0.05, family- wise error (FWE)-corrected for multiple comparisons. In the opposite contrasts (acceptance > passive viewing; suppression > passive viewing), whole-brain analyses did not result in significant effects.

### 3.2. Functional imaging findings

#### 3.2.1. Neural activation during emotion acceptance

Functional brain imaging results are shown in Tables 2, 3. In BPD patients, whole-brain analyses revealed decreased activation during emotion acceptance compared to the passive viewing condition (deactivation). Analyses identified a significant cluster (*k* = 135 voxels) with a deactivation peak in the right insula. For the opposite contrast (emotion acceptance > passive viewing), no significant results were found. In HC, whole-brain analyses did not reveal significant activation differences between emotion acceptance and passive viewing. Activation differences related to emotion acceptance between BPD patients and HC were not found either.

**TABLE 3:** Results of the regions of interest (ROI) analyses indicating acceptance-related bilateral insular underactivation and suppression-related right caudate overactivation in subjects with borderline personality disorder (BPD) during the emotion regulation task.

ROIs	MNI coordinates			Cluster size		
	*x*	*y*	*z*	*k*	*t*	*p*
**Acceptance (acceptance > passive viewing)**						
**HC > BPD**						
Right insula	45	–7	4	7	3.78	0.030
Left insula	–39	5	1	2	3.44	0.040
**BPD > HC**						
No significantly activated voxels.						
**Suppression (suppression > passive viewing)**						
**HC > BPD**						
No significantly activated voxels.						
**BPD > HC**						
Right caudate	18	–16	25	1	3.41	0.044

BPD, borderline personality disorder; HC, healthy control; *t*, *t*-value, *k*, cluster size (in voxels). Regions of interest (ROI) results were tested at the voxel level with a significance threshold of *p* < 0.05, family- wise error (FWE)-corrected for multiple comparisons. In the opposite contrasts (acceptance > passive viewing; suppression > passive viewing), ROI analyses did not result in significant effects.

**FIGURE 2:**
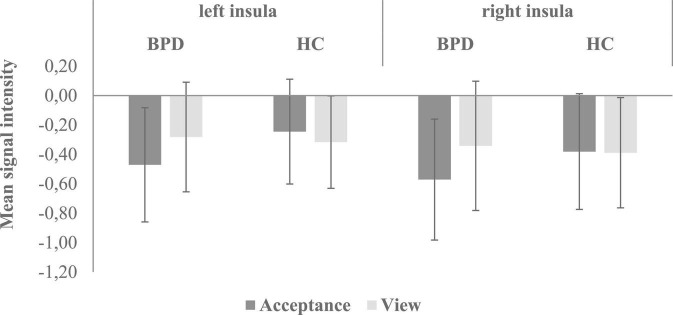
Mean signal intensity (beta-weights) in the left and right insula during emotional acceptance and passive viewing in BPD patients and HC. There was a significant emotion regulation condition × group interaction (*F*_1,42_ = 9.58, *p* = 0.003, η^2^ = 0.186), indicating a stronger acceptance-related deactivation in the BPD group compared to the HC. BPD, borderline personality disorder; HC, healthy controls.

Regions of interest analyses revealed higher bilateral insular activation in HC than in patients with BPD during emotion acceptance (acceptance > passive viewing). In the other ROIs (bilateral amygdala, hippocampus, ACC, caudate, putamen, DLPFC, VLPFC) and for the opposite contrast (acceptance < passive viewing), no significant group differences were found. Figure 2 shows the mean signal intensity in the left and right insula during emotional acceptance and passive viewing in BPD patients and HC. Repeated measures ANOVA involving left and right insular signal intensity revealed a significant emotion regulation condition × group interaction (*F*_1,42_ = 9.58, *p* = 0.003, η^2^ = 0.186), indicating a stronger acceptance-related deactivation in the BPD group compared to the HC. Furthermore, signal intensity was lower during emotion acceptance compared to passive viewing (main effect of emotion regulation condition: *F*_1_,_42_ = 4.46, *p* = 0.041, η^2^ = 0.096) and also lower in the right than in the left hemisphere (main effect of hemisphere: *F*_1_,_42_ = 21.64, *p* < 0.001, η^2^ = 0.340). By contrast, the main effect of group was not significant at *p* < 0.05.

#### 3.2.2. Neural activation during emotion suppression

In BPD patients, whole-brain analyses revealed decreased activation during emotion suppression compared to the passive viewing condition (deactivation). Analyses identified a significant cluster (*k* = 551 voxels) with deactivation peaks in the right cerebellum and the left calcarine. For the opposite contrast (emotion suppression > passive viewing), no significant results were found. In HC, whole-brain analyses did not reveal any significant activation differences between emotion suppression and passive viewing. Activation differences related to emotion suppression between BPD patients and HC were not found either.

Regions of interest analyses revealed higher right caudate activation in BPD compared to HC during emotion suppression (suppression > passive viewing). In the other ROIs (bilateral amygdala, insula, hippocampus, ACC, putamen, DLPFC, VLPFC) and for the opposite contrast (suppression < passive viewing), no significant group differences were found. Repeated measures ANOVA involving right caudate signal intensity) did not reveal significant mean or interaction effects at *p* < 0.05.

## 4. Discussion

In the current imaging study, we investigated neural activation during instructed emotional acceptance and suppression in patients with BPD and healthy subjects. As expected, both groups showed considerable activation differences related to active emotion regulation. With regard to emotion acceptance, HC demonstrated stronger activation than BPD patients in the bilateral insula (acceptance > passive viewing), probably reflecting a decreased insular participation in patients with BPD while engaging in emotional acceptance. Moreover, participants with BPD showed greater activation than HC in the right caudate during emotion suppression (suppression > passive viewing). Noteworthy, we did not observe any further ROI activation differences.

Emotional dysfunctions and altered neural activities during emotion regulation have been frequently associated with BPD (18). However, in contrast to previous research [see for an overview (33)], our study did not address a primarily cognitive approach to modify emotion experience, such as cognitive reappraisal. Instead, we focused on a more experiential approach for dealing with emotional states, namely emotion acceptance and suppression. We provide evidence for a hypoactive insula in BPD patients when instructed to accept their emotions in response to fearful movie clips. An increasing number of neuroimaging studies point toward the relevance of the insula for mindfulness practice (34) that includes the acceptance of present experiences such as emotions. The insula, especially the anterior part, is known to play an integral part in processing interoceptive information important for inner emotional awareness (35, 36). As emotion acceptance requires detachment, attention, and emotional awareness (34), altered insular functioning may reflect a reduced interoceptive sensitivity and a dysregulated appraisal of internal experience in BPD (37). Accordingly, emotion dysfunction in BPD has been frequently linked to a lack of emotional awareness and emotional clarity (38). Furthermore, previous research provides some support for altered insula activation in individuals with BPD when being confronted with negative compared to neutral stimuli [e.g., (39, 40)]. In addition, improvement in BPD symptoms after DBT was associated with changes in insular activity (41), which may be interpreted as a beneficial effect of becoming familiar with emotion acceptance. However, most studies found therapy-related changes to be associated with anterior insula functioning in BPD (41, 42), whereas in our study, it is mainly the posterior part of the insula that is affected by emotion acceptance.

Another finding of our study was that BPD patients demonstrated an elevated caudate activity when instructed to suppress fear, which confirms previous results of our working group linking striatal activation to reduced emotion acceptance in BPD (21). Taken together, these data indicate that striatal activation in BPD is linked to both increased emotion suppression and reduced emotion acceptance, respectively. Of note, even though there is a growing body of literature that recognizes the critical role of the striatum in affective processes [e.g., (43)], very little attention has been paid to the role of striatal functioning in processing negative affect in BPD. An integrative review on the neural processes in healthy individuals suggested that striatal areas are particularly involved in motor programs activated by unpleasant stimuli and associated with emotional expressions or withdrawal behaviors (44). In general, previous research emphasizes the role of the caudate in well-learned and habitual motor and cognitive processes [e.g., (45)]. Considering previous data on the habitual use of emotion regulation strategies in BPD (2), our results may reflect the well-learned preference of BPD patients to engage in emotion suppression.

Debate continues about the putative benefit of emotion acceptance but also of emotion suppression in BPD individuals (15). Indeed, the broad and heterogeneous conceptualization of both strategies offers a plausible explanation for the existing contradictions. However, some authors further suggested that acceptance-based strategies may require a longer training period than other regulation strategies to be effective (46). Following this argumentation, previous research indicated that insula activity during mindfulness meditation is linked to the level of prior meditation experience (47), which has been interpreted as an indicator for the learnability of mindfulness and acceptance-based skills, respectively. Future studies should therefore investigate emotion acceptance and suppression before and after interventions aiming at improving emotion regulation ability in BPD to further elucidate the neural processes after developing a more habitual and expert use of emotion acceptance.

The current study offers some important findings on the neural substrates of emotion acceptance and suppression, both of which represent clinically relevant targets for emotion dysregulation treatments in BPD. However, a number of limitations have to be discussed. First, the major limitation of the current approach is the lack of an objective measure that could have served as manipulation check. Hence, we cannot confirm whether participants actually complied with the instructions to regulate their emotion experience as intended. However, we used previously validated film clips to induce fear and all participants were carefully trained in the respective emotion regulation strategy during the pre-session procedures to ensure that they were able to follow the emotion regulation instructions. Likewise we did not directly access the effects of emotional acceptance and suppression on self-reported emotional experience in order to avoid confounding interferences due to executive and evaluative processes. As a result, conclusions about effects on subjective emotional experiences remain speculative to some extent. Second, the purpose of this research was the exploratory investigation of activation differences between BPD and HC when applying emotion acceptance and suppression. Surprisingly, we found significant activation differences in both conditions only in small clusters. However, it has to be taken into account that we focused on two broad emotion regulation strategies with high ecological validity, but likely based on a number of different sub-processes (e.g., monitoring and attentional control). Future research with respect to emotion acceptance and suppression should include the respective sub-processes. Third, the study results are limited to the processing of fearful face stimuli. As research recently has shown emotion regulation strategies to exhibit emotion-specific but also emotion-invariant effects (48), future research should include other emotional stimuli such as anger-related stimuli. Fourth, we cannot rule out the possibility that methodological factors such as the short training duration influenced the study results. Future research should address the conscious application of emotion acceptance and suppression before and after an extensive acceptance-based treatment in order to capture not only temporary benefits but also sustained effects on neural processes after becoming familiar with emotion acceptance.

Our study offers some new insights into the neural mechanisms of emotion acceptance and suppression. We showed that the conscious application of emotion acceptance reduced insular activation in individuals with BPD, which corresponds with their difficulties in identifying and being aware of emotion experiences. In contrast, emotion suppression enhanced caudate activation in BPD. This finding may support the assumption that striatal activation may index processes associated with well-learned cognitive routines in BPD such as emotion suppression.

## Data availability statement

The raw data supporting the conclusions of this article will be made available by the authors, without undue reservation.

## Ethics statement

The studies involving human participants were reviewed and approved by University of Münster, Ethics Committee, Germany. The patients/participants provided their written informed consent to participate in this study.

## Author contributions

SF: conceptualization, methodology, software, project administration, data curation, formal analysis, and writing – original draft preparation. TB: conceptualization, methodology, funding acquisition, and writing – reviewing and editing. AL: project administration, software, investigation, and writing – reviewing and editing. NS: conceptualization, funding acquisition, and investigation. FW: resources and supervision. MD: conceptualization, resources, and writing – reviewing and editing. MT: conceptualization, methodology, funding acquisition, software, validation, and writing – reviewing and editing. All authors contributed to the article and approved the submitted version.

## Footnotes

1
https://www.fil.ion.ucl.ac.uk/spm/software/spm12/
